# Cognitive failures in late adulthood: The role of age, social context and depressive symptoms

**DOI:** 10.1371/journal.pone.0189683

**Published:** 2017-12-15

**Authors:** Paul Kenneth Hitchcott, Maria Chiara Fastame, Dalila Langiu, Maria Pietronilla Penna

**Affiliations:** Department of Pedagogy, Psychology, Philosophy, University of Cagliari, Cagliari, Italy; Kobenhavns Universitet, DENMARK

## Abstract

The incidence of self-reported cognitive failures among older adults may be an index of successful cognitive aging. However, self-reported cognitive failures are biased by variation in depressive symptomatology. This study examined age-related and socio-cultural context effects on cognitive failures while controlling for depressive symptoms. Both overall and specific factors of cognitive failures were determined. A further goal was to investigate the relationship between working memory and cognitive efficiency measures and cognitive failures. One hundred and thirty-nine cognitively healthy adults were recruited from two populations known to differ in their dispositions toward cognitive failures and depressive symptoms (Sardinia and northern Italy). The participants were assigned to Young Old (65–74 years old), Old (75–84 years of age) or Oldest Old (≥85 years of age) groups, and individually presented with a test battery including the Cognitive Failures Questionnaire, the Centre for Epidemiological Studies of Depression Scale, and Forward and Backward Digit Span tests. Specific factors of cognitive failures were differentially associated with measures of depression and working memory. While age had no impact on any aspect of cognitive failures, overall and specific dispositions varied between the two populations. The overall liability to cognitive failure was lower in participants from Sardinia, however, this group also had a higher liability to lapses of action (Blunders factor). Overall, these findings highlight that richer information about cognitive failures may be revealed through the investigation of specific factors of cognitive failures. They also confirm that the absence of changes in cognitive failures across old age is independent of variation in depressive symptoms, at least among cognitively healthy elders.

## Introduction

Cognitive failures are unintended errors in normally competent everyday behaviours that are frequently minor, albeit irritating (e.g., locking keys into your car), but may at times interfere with the completion of routine activities and even result in serious accidents or injuries [1]. Cognitive failures are typically assessed by self-report and several questionnaires have been developed for this purpose, the most well-known being the Cognitive Failures Questionnaire (CFQ)[2]. This measure captures a broad range of commonplace cognitive failures: memory slips (e.g., forgetting where something has been left), perception slips (e.g., failing to notice road signs) and action slips (e.g., bumping into people). CFQ scores exhibit good test-retest reliability over periods up to 2 years [3] and correlate significantly with other comparable constructs [4]. Such findings have led to widespread acceptance that the CFQ indexes variability in a disposition toward cognitive failures.

Widely held negative stereotypes frequently characterize older people as forgetful, absent-minded and bumbling i.e., more prone to cognitive failures [5]. However, although several studies have explored the correlation between ageing and cognitive failures, a clear picture has not emerged. Reports of increases [6, 7], decreases [8, 9, 10] and no differences [11, 12] are available. Closer inspection of study methodology does not suggest that any single factor explains the observed relationship between ageing and cognitive failures, however, one consistent limitation is a failure to control for the impact of depressive symptoms which known to be positively correlated with cognitive failures [7, 13]. Depressive symptomology is likely to distort the relationship between ageing and cognitive failures because levels vary with age. Previous reports have described a U-shaped function with levels only reaching the levels seen in young adulthood after the age of 75 [14, 15]. Controlling for depressive symptomatology would therefore appear to be essential for reliable determination of age-related changes in cognitive failures.

A further complication is that ageing may be associated with increases in only certain types of cognitive failure. The most convincing support for this view was reported by Rast, Zimprich, Boxtel and Jolles [16] who examined changes in 3 components of the CFQ in 1000 participants aged 24–83 years. Ageing was associated with a near linear increase in forgetfulness, decreased distractibility in those >64 years, and no significant changes in false triggering. This evidence confirms and extends earlier indications of a differential impact of aging on individual CFQ item scores [17]. In addition, it highlights that complex changes may be present masked if represented in terms of a simple total score. However, no prior research has investigated whether age-related changes in specific CFQ subscales or items are confounded by depressive symptoms.

Such an investigation may also be advantageous with respect to the inconsistent literature concerning the association between cognitive failures and objective measures of cognitive function. Much attention has been given to the fact that subjective cognitive complaints correlate weakly, if at all, with objective measures of cognition [18]. This situation extends to studies that have employed the CFQ as a measure of subjective cognitive complaints. Thus, total CFQ scores have been found to be correlated with objective indices of cognitive performance in some [19], but not all [7] studies, even after controlling for depressive symptoms. Whether greater consistency would be found by examining specific CFQ factors has not yet been reported. In addition, high total CFQ scores are more reliably correlated with vulnerability to future objective decline [19]. Low total CFQ scores may represent an antecedent of successful (cognitive) ageing [20] and are a known to be a characteristic of the longevous population inhabiting the central eastern region of Sardinia [21]. How this attribute is represented at the level of specific CFQ factors and whether it is confounded by depressive symptomatology is unknown. Significantly, older people from the Sardinian Blue Zone are known to have substantially lower levels of depressive symptomatology than do older people from outside this region [22, 23].

The overall goal of the present study was to provide richer and more reliable detail about cognitive failures in old age. The specific aims of the present study may be summarized as follows: 1) confirm previous reports [21, 22] of fewer depressive symptoms and total cognitive failures in older people from Sardinia relative to a control sample from northern Italy; 2) conduct a detailed examination of the effects of social context on cognitive failures (total and factor scores) during late adulthood, while controlling for depressive symptoms; 3) determine the associations between cognitive failures (total and subscale scores) and objective measures of working memory and general cognitive efficiency. In relation to these aims the following hypotheses can be stated: 1) the Sardinian sample will report fewer total cognitive failures and depressive symptoms than the control sample; 2) depressive symptoms will be significantly correlated with cognitive failures and the effect of social context on cognitive failures will be reduced after controlling for differences in depressive symptoms; 3) cognitive failures will be unrelated to objective measures of working memory and general cognitive efficiency.

## Materials and methods

### Participants

One hundred and thirty-nine cognitively healthy community-dwelling adults participated voluntarily in the current study. Consistent with similar previous research [24, 25] participants were assigned to one of 3 age groups: Young Old (65–74 years old), Old (75–84 years of age) or Oldest Old (≥85 years of age). Participants were recruited from 2 different geographical regions of Italy via personal contacts, direct appeals to local community groups and through liaison with the local municipalities which provided lists of residents over 64 years old. Specifically, seventy participants were recruited from the village of Tempio Pausania, which is located in a mountainous region of northern Sardinia. Sixty-nine participants recruited from rural areas of Lombardy, in Northern Italy served as a control group. Various steps were taken to minimize the risk that the samples differed with respect to potential confounding characteristics. First, both groups were recruited from rural areas similarly characterized by a predominantly agro-pastoral lifestyle. While the available data suggest that these two areas are broadly comparable in terms of many economic, social and health outcomes [26] details for several possible demographic influences were collected for statistical confirmation. As shown in Table 1 the two region groups did not differ in terms of gender (χ^22^ = .32, df = 3, p = .85), education (*i*.*e*., 0–8 years versus > 8 years) (χ^2^ = .32, df = 2, p = .85), widowhood (χ^2^ = .35, df = 1, p = .55), daily medicinal usage (i.e., yes versus no, χ^2^ = .07, df = 1, p = .78) and involvement in either social leisure activities/hobbies (i.e., yes versus no, χ^2^ = 2.63, df = 1, p = .10) or gardening (i.e., yes versus no, χ^2^ = .009, df = 1, p = .93). Furthermore, across the three age groups, the distribution of people living alone (χ^2^ = .84, df = 2, p = .66) or with the spouse (χ^2^ = .8451, df = 2, p = .77) or with children (χ^2^ = 1.96, df = 2, p = .38) was similar. Finally, although more Sardinians lived with a helper than did participants recruited in Northern Italy (χ^2^ = 6.6, df = 2, p = .04), the proportion of such individuals low in both regions. Table 1 summarizes these details.

**Table 1:** Socio-demographic characteristics for participants of the study split by age group and social context. Total, mean and standard deviation (in parentheses) where appropriate.

		Young Old Group	Very Old Group	Oldest-Old Group
		(65–74 years)	(75–84 years)	(> 84 years)
**Sardinia**	**Total N**	23	24	23
	**Gender**			
	**Males**	12	12	11
	**Females**	11	12	12
	**Age (years)**	70.0	78.6	88.0
		(2.8)	(2.9)	(3.0)
	**Education (years)**			
	≤ 8	11	12	12
	> 8	12	12	11
	**Widowhood**			
	Yes	6	12	14
	No	17	12	9
	**Living with**			
	no one	3	7	6
	spouse	18	12	10
	children	1	3	1
	helper	1	2	6
	**Medicines**			
	Yes	20	19	21
	No	3	5	2
	**Hobbies**			
	Yes	12	12	9
	No	11	12	14
	**Gardening**			
	Yes	12	11	9
	No	11	13	14
**Northern**				
**Italy**	**Total N**	21	24	24
	**Gender**			
	**Males**	12	12	12
	**Females**	9	12	12
	**Age (years)**	70.8	78.8	87.4
		(2.4)	(2.8)	(2.4)
	**Education (years)**			
	≤ 8	9	12	12
	> 8	12	12	12
	**Widowhood**			
	Yes	7	11	17
	No	14	13	7
	**Living with**			
	no one	4	10	15
	spouse	15	13	7
	children	0	1	2
	helper	2	0	0
	**Medicines**			
	Yes	13	23	22
	No	8	1	2
	**Hobbies**			
	Yes	15	14	13
	No	6	10	11
	**Gardening**			
	Yes	13	10	8
	No	8	14	16
**Total per age group**		44	48	47

Furthermore, all participants had to satisfy the following inclusion criteria to take part: 1) to have been born in the areas where they were recruited and currently reside thereof; 2) be a descendant of individuals also resident in that area for at least two previous generations; 3) be cognitively intact as assessed by the Mini-Mental State Examination, i.e., and age- and education-adjusted score score ≥ 24 [27]. The first criterion rules out migrations bias [28] while the second ensures that the validity of self-report measures is not compromised by cognitive impairment [29]. The final total sample was similar both in size and composition to those employed in previous studies that have successfully demonstrated the existence of distinct psychological characteristics in the Sardinian blue zone population [21–23, 25]. No incentives were offered in return for participation.

### Ethics statement

The ethical committee of the Department of Pedagogy, Psychology, Philosophy of the University of Cagliari approved this study. Written informed consent was given by all participants prior to participation.

### Materials

Each participant was completed the following inventories:

1. The Mini-Mental State Examination (MMSE)[27]. This instrument includes 30 items assessing various cognitive functions, such as short and long-term memory, spatial-temporal orientation, attention, motor coordination, mental calculation and language. Participants scoring <24/30 were excluded due to suspected cognitive decline. Individual scores were corrected both for age and education, in agreement with previous research conducted in Italy [30].
2. The preliminary interview developed by Fastame and Penna [25] was used to collect basic socio-demographic characteristics (e.g., age, marital status, years of education) for each participant.
3. The Centre for Epidemiological Studies of Depression Scale (CES-D) [31], Italian version [32]. This is a 20-item self-report inventory assessing the frequency of depressive signs (e.g., guilt, loss of interest, fatigue, sadness) experienced over the past week on a four-point Likert scale (from 0, *never or rarely* to 3, *most days or every day*). Item responses are totaled yielding a single score (maximum = 60). Previous research in Italian samples has demonstrated that scores of 16–23 identify individuals at risk for mild-moderate depression, whereas scores ≥24 indicates a risk for severe depression [32]. We observed good internal consistency concerning this measure–Cronbach’s alpha = .84.
4. The Italian version of the CFQ [2, 33]. This instrument consists of 25 items assessing the frequency of all types of CF (perceptual, memory, action) in the daily life during the past six months on a five-point Likert scale ranging from 0 (i.e., *never*) to 4 (i.e., *very often*). A total score across all 25 items provided a measure of the general liability to cognitive failures, while four factor scores used to examine the liability to specific types of cognitive failure [4, 34]. These are derived from the three categories of common errors (lapses of memory, attention and action) identified by Broadbent et al., [2] but with further partitioning of the memory component. The resulting four subscales are: (1) Memory–an index of general memory failures and forgetfulness (e.g., forgetting where something was left); (2) Distractibility–an index of failures related to failures of perception and attention (e.g., reading something without thinking about it); (3) Blunders–an index of slips of action or physical mishaps (e.g. bumping into someone); (4) Memory for Names–an index of failures related to people’s names (e.g., forgetting someone’s name). Consistent with previous research [4, 34], we observed good internal consistency with respect to the total CFQ score (Cronbach’s alpha = .83). Because each of the four factor scores were derived from relatively few items (<10) internal consistency was assessed in terms by calculating mean inter-item correlation coefficients [35]. Values for each of the four subscales were as follows: Memory .20; Distractibility .15; Blunders .20; Memory for Names .61. The first three values lie within either the optimal (i.e., .20-.40) or moderate (i.e., < .20) range of values [35]. The high value for Memory for Names is consistent with this factor being derived from only 2 items, both related to the same specific cognitive failure.
5. Forward Digit Span Test [33, 36] is an objective measure of the passive component of the verbal sequential working memory component. The participant is asked to recall a sequence of numbers in the same presentation order immediately after sequence presentation. Sequences of increasing length are presented until the participant fails two sequences having the same length. Span is calculated as the average of the two longest sequences correctly recalled by the participant.
6. Backward Digit Span Test [33, 36] is an objective measure of active verbal sequential working memory processes. Participants are required to repeat, in the reverse order, digit strings immediately after their presentation, first recalling the last digit in the sequence presented, then the penultimate digit, and so on. The task was administered and scored in exactly the same manner as the Forward Digit Span Test.

### Procedure

All participants were tested individually in their own homes. After explaining the general aim of the study participants provided written informed consent. Following international guidelines on the conduct of research in older adults, the absence of impairment on measures of neurocognitive function was the key criterion used to assess consent capacity in the current sample [37]. Thus, even though there is evidence that some dimensions of consent capacity (e.g., making the reasonable choice and expressing choice) are preserved even in individuals with mild and moderate Alzheimer’s dementia [38–39], capacity to consent was first determined in terms of cognitive status using the Mini Mental State Examination. In addition, comparable with similar previous research [40], we determined two further criteria via preliminary interview [21]. These confirmed the absence of 1) any physical disease or condition that could potentially affect cognition (such as cerebrovascular or other neurologic diseases); and 2) any psychiatric condition that could affect capacity to consent.

To reduce the risk of fatigue among the participants, the experimenter read aloud individual inventory items and recorded the spoken responses. All participants first completed the MMSE test, followed by the socio-demographic interview. After that, the presentation order of the CES-D, CFQ and working memory tests was counterbalanced across participants. Each experimental session lasted about 50 minutes.

### Statistical analyses

Initially, two separate analyses were conducted to examine whether the two independent variables (age group and socio-cultural context) affected the primary outcomes, cognitive failures and depressive symptomatology. Multivariate analysis of variance [41] was used to assess between-group differences in cognitive failures (including the CFQ total and subscale scores as dependent variables). Analysis of variance [41] was used to assess between-group differences in depressive symptoms. In both cases, group differences were compared across 3 age group levels—young old (65–74years), very old (75–84 years), oldest old (>84 years) and across 2 levels of socio-cultural context—Sardinia *vs*. northern Italy. As the main effects of age group and socio-cultural context were significant both for cognitive failures and depressive symptoms, a subsequent multivariate analysis of covariance [41] was conducted to determine whether the observed differences in cognitive failures (including the CFQ total and subscale scores as dependent variables) were independent of covariation in depressive symptoms. Where appropriate, post-hoc comparisons using Tukey’s Honestly Significant Difference test are reported.

The remaining aim of the investigation, the examination of the relationships between cognitive failures (CFQ total and subscale scores) and objective indices of cognitive performance, was assessed via a series of bivariate partial correlations (controlling for covariance in depressive symptoms). The objective measures of cognitive performance were MMSE score, forward and backward digit span. Age was included to confirm the presence of age-related decline in objective cognitive performance.

## Results

Table 2 illustrates the mean scores in CFQ, CES-D and working memory measures across the three age groups living in Sardinia and Northern Italy, respectively.

**Table 2:** Mean scores (with standard deviations in parentheses) on the cognitive failure questionnaire and its subscales, the CES-D, forward and backward digit span tests.

	Young Old Group (65–74 years)	Very Old Group (75–84 years)	Oldest-Old Group (>84 years)
**Sardinia**			
CFQ—Total	29.5 (11.6)	27.1 (14.8)	28.7 (10.5)
CFQ—Memory	6.7 (3.4)	6.1 (3.7)	6.3 (3.4)
CFQ—Distractibility	11.4 (5.1)	12.3 (5.5)	14.2 (3.9)
CFQ—Blunders	9.7 (4.6)	8.2 (4.8)	8.1 (4.2)
CFQ—Names	2.3 (2.0)	2.1 (1.7)	2.6 (1.9)
CES-D	6.3 (5.7)	8.0 (7.3)	10.0 (8.1)
Forward Digit Span	4.8 (1.2)	5.4 (1.5)	5.1 (1.7)
Backward Digit Span	4.2 (1.3)	4.2 (1.3)	3.8 (0.9)
**Northern Italy**			
CFQ—Total	32.2 (16.3)	41.4 (11.4)	37.1 (15.0)
CFQ—Memory	8.2 (5.4)	10.4 (4.9)	9.9 (5.7)
CFQ—Distractibility	14.0 (6.1)	18.4 (4.9)	16.2 (5.7)
CFQ—Blunders	5.7 (4.5)	6.2 (4.1)	5.5 (3.4)
CFQ—Names	4.2 (2.3)	6.3 (2.1)	5.4 (2.1)
CES-D	17.3 (10.9)	26.9 (12.5)	22.2 (9.9)
Forward Digit Span	7.4 (3.2)	6.1 (2.5)	4.1 (1.8)
Backward Digit Span	4.6 (2.1)	3.9 (1.6)	2.6 (1.5)

In order to pursue the first aim, a 2 (socio-cultural context: Sardinia versus Northern Italy) X 3 (age group: Young Old, Very Old and Oldest-Old) Multivariate Analysis of Variance (MANOVA) was conducted to investigate the impact of age-related factors and socio-cultural context on total CFQ, Distractibility, Blunders and Memory for Names scores. The multivariate tests documented the significant main effects of the socio-cultural context (Wilks’ ⋀ = .41, df = 4;130, p < .0005), age group (Wilks’ ⋀ = .85, df = 8;262, p = .007). The main effect of the socio-cultural context was significant for total CFQ [F(1,133) = 9.2, p < .003, ηp^2^ = .06], Memory [F(1,133) = 16.7, p < .0005, ηp^2^ = .11], Distractibility [F(1,133) = 16.2, p < .0005, ηp^2^ = .11], Blunders [F(1,133) = 15.4, p < .0005, ηp^2^ = .10] and Memory for Names [F(1,133) = 75.2, p < .0005, ηp^2^ = .36]. Sardinians reported less failures in all the measures than peers living in northern Italy, except that in the Blunder subscale, where the former group reported higher scores. The main effect of age group was significant only for the Distractibility index [F(2,133) = 3.6, p = .03, ηp^2^ = .05]. Tukey’s post-hoc comparisons revealed that Young Old participants reported lower Distractibility (M = 12.7, SD = 5.7) than the Very-Old group (M = 15.4, SD = 6, p = .04). A difference approaching significance was found between the Young-Old and the Oldest-Old (M = 14.4, SD = 5.6, p = .054) groups.

There was little indication that the relationship between age and cognitive failures varied between the two social contexts. Only the interaction between socio-cultural context and age group was significant only for the Memory for Names score [F(2,133) = 3.7, p = .03, ηp^2^ = .05]. In order to further explore this interaction, a series of ANOVAs showed that 65–74 years old participants from Lombardy reported more memory mistakes (M = 4.2, SD = 2.3) than Sardinian peers (M = 2.3, SD = 2.03) [F(1,42) = 8.6, p = .005, ηp^2^ = .17]. A similar socio-cultural effect was also found across the Very-Old [F(1,46) = 58.9, p < .0005, ηp^2^ = .56] and Oldest-old [F(1,45) = 23.3, p < .0005, ηp^2^ = .34] groups.

Then, in order to achieve the second goal, an Analysis of Variance (ANOVA) was conducted to explore the effect of age-group and socio-cultural context on the CES-D measure. This revealed the significant main effects of socio-cultural context [F(1,133) = 77.7, p < .0005, ηp^2^ = .37] and age group [F(2,133) = 4.6, p = .01, ηp^2^ = .06], whereas the interaction between age group X socio-cultural context was not significant [F(2,133) = 2.4, p = .1]. Overall, Sardinians reported less depressive signs (M = 8.1, SD = 7.2) than peers residing in northern Italy (M = 22.3, SD = 11.7). Moreover, Tukey’s post-hoc comparisons revealed that Young Old were significantly less depressed (M = 11.5, SD = 10.1) than Very Old (M = 17.4, SD = 14, p = .008) and Oldest-Old (M = 16.2, SD = 10.9, p = .04).

The third aim was achieved conducting a 2 (socio-cultural context: Sardinia versus Northern Italy) X 3 (age group: Young Old, Very Old and Oldest-Old) Multivariate Analysis of Covariance (MANCOVA) that was carried out to investigate the impact of age-related factors and socio-cultural context on Memory, Distractibility, Blunders and Memory for Names scores, controlling for the effect of the covariate CES-D. The multivariate tests documented the significant main effects of the socio-cultural context (Wilks’ ⋀ = .52, df = 4;129, p < .0005), age group (Wilks’ ⋀ = .87, df = 8;258, p = .02) and depression score (Wilks’ ⋀ = .87, df = 4;129, p = .001). The main effect of the socio-cultural context was significant for Blunders [F(1,132) = 17.13, p < .0005, ηp^2^ = .11], and Memory for Names [F(1,132) = 47.3, p < .0005, ηp^2^ = .26] but not for Memory [F(1,132) = 1.49, p = .22] and Distractibility [F(1,132) = 1.36, p = .24] indexes. Overall, Sardinians reported fewer Memory for Names failures (M = 2.3, SD = 1.9) than elderly people from northern Italy (M = 5.3, SD = 2.3), but more Blunders (M = 9.2, SD = 4.5) than the group enrolled in northern Italy (M = 5.3, SD = 4).

The main effect of age group was not significant for any of the CFQ scores [F(2,132) = ranging from .002 to 2.5 p >.05]. In contrast, the main effect of the covariate CES-D was significant on Memory [F(1,132) = 12.7, p = .001, ηp^2^ = .09] and Distractibility [F(1,132) = 12.7, p < .0005, ηp^2^ = .07] scores but not in Blunders [F(1,132) = 2.7, p = .1] and Memory for Names [F(1,132) < .0001, p = .98] conditions. Finally, the interaction between socio-cultural context X age group was significant only for Memory for Names [F(2,132) = 3.6, p = .03, ηp^2^ = .05]. The same pattern of results previously described above for the MANOVA was found, therefore, for brevity it will not be repeated here.

Furthermore, as reported in Table 3, associations between objective measures of cognition and self-reported CF were explored via a series of partial correlations (summarized in Table 2). These indicated that Distractibility (r = -.20, p < .05) and Blunders (r = -.21, p < .05) were significantly correlated with Forward Digit Span respectively, whereas Blunders correlated only with Forwards Working Memory Span. Moreover, age was significantly correlated with Forward (r = -.28, p<0.001) and Backward (r = -.31, p < .0005) digit span. No further significant correlations were found among any objective measure of cognitive performance (i.e., Forwards and Backwards Digit Span, or MMSE) and the various measures of cognitive failures.

**Table 3:** Patterns of partial correlations between cognitive failures, working memory, cognitive efficiency and age.

	*CFQ Memo*	*CFQ Distr*	*CFQ Blun*	*CFQ Names*	*Forw Digit*	*Back Digit*	*MMSE*	*Age*
*CFQ total*	.84***	.87***	.72***	.60***	-.15	-.10	-.05	.01
*CFQ Memo*	1	.66***	.47***	.41***	-.10	-.08	-.07	.01
*CFQ Distr*		1	.50***	.46***	-.19*	-.13	-.01	.13
*CFQ Blun*			1	.20*	-.21*	-.08	-.06	-.09
*CFQ Names*				1	.06	-.04	-.03	.07
*Forw Digit*					1	.55***	.30***	-.28**
*Back Digit*						1	.31***	-.31***
*MMSE*							1	.02
*Age*								1

Abbreviations: total CFQ score (CFQ total); CFQ Memory subscale (CFQ Memo); CFQ Distractibility subscale (CFQ Distr); CFQ Blunders subscale (CFQ Blun); CFQ Memory for Names subscale (CFQ Names); Forward Digit Span (Forw Digit); Backward Digit Span (Back Digit); Mini-mental state examination score (MMSE).

***p < .0001

**p < .01

*p < .05

## Discussion

There is marked inconsistency concerning the impact of ageing on cognitive failures, errors in normally competent everyday task performance [2, 17]. This may be because only certain types of failures increase in old age and because of a confounding influence of depressive symptomatology, issues that have received only limited attention [16, 42]. To address this weakness in the literature, a detailed examination of the liabilities to specific types of cognitive failure (using CFQ factor scores based on [4]) was made in tandem with measurement of depressive symptoms, allowing for statistical control of the latter. Cognitive failures were examined in two populations of older people known to differ in terms of their overall disposition to cognitive failure [21]. The relationship between the specific types of cognitive failure and objective measures of cognitive function was also investigated.

Overall, the current findings are consistent with previous reports [16, 42] indicating that the examination of distinct factors of cognitive failures can be advantageous. Initial analyses confirmed that total CFQ scores were lower in Sardinian participants relative to a control group from northern Italy [21], however, differential changes in specific types of cognitive failure were revealed when CFQ factor scores were examined. Thus, compared to peers living in Northern Italy, Sardinian participants had a reduced liability to 3 types of cognitive failure—Memory, Distractibility and Memory for Names. In contrast, levels of the fourth factor, Blunders, were higher in Sardinian participants. We also investigated whether these differences were independent of variation in depressive symptomatology. As expected [23, 43] Sardinian participants reported much lower depressive symptomatology than those from Northern Italy. After statistically controlling for the impact of depressive symptoms, the reduced liabilities to failures of Memory and Distractibility in Sardinian participants were lost. In contrast, the differences in Blunders (i.e., higher in Sardinians) and Memory for Names (i.e., lower in Sardinians) remained significant.

To the best of our knowledge these findings are the first demonstration that depressive symptoms are differently correlated with specific types of cognitive failures. Depressive symptomatology was a significant covariate of only two of the four factors–Memory and Distractibility. These factors may therefore contribute more to the known association between total CFQ scores and increased vulnerability to stress and related measures such as neuroticism, trait anxiety and depressive symptomatology [2, 42, 44]. By contrast, depressive symptoms were not significantly correlated with either Blunders or Memory for Names. No certain explanation for the increased liability to Blunders in Sardinian elders is available. However, one possibility is that it might be related to differences in physical activity levels. In young adults, the liability to Blunders is positively correlated with stimulation seeking and a causal link whereby higher physical activity increases the risk of such mishaps occurring is indicated [34]. If this relationship persists into late adulthood it could account for the observed increase in Blunders, since Sardinian elders are known to be more physically active than their counterparts from northern Italy [45]. Why Sardinian participants reported fewer Memory for Names failures is less clear, but might be related to the prevailing tight-knit, highly sociable culture of the region [22].

While self-reported cognitive failures varied between the two social contexts, there was very little evidence that they varied across old age despite the broad age range of participants. The only suggestion (Distractibility factor) was lost after statistically controlling for differences in depressive symptoms. In addition, all but one of the interactions involving age and social context were non-significant, the single exception being the Memory for Names factor. While several studies have found that total CFQ scores do not increase, or even decline slightly with age [see 17], none have demonstrated that this relationship is independent of variation in depressive symptoms. The present findings confirm this to be the case even at the level of specific CFQ factors. One interpretation of this finding is that aging is associated with diminishing awareness of actual cognitive decline. However, a lack of correspondence between CFQ scores and objective measures of cognitive performance is well-documented at all ages, as is the stronger association with indices of negative affect [17]. It should also be recognized that the present report is one of relatively few studies to have examined cognitive failures thoroughly in adults aged over 85 (i.e., the oldest-old). The absence of age-related increases in cognitive failures is particularly striking in this respect since this group report the highest levels of subjective cognitive complaints and exhibit the greatest decrements in objective cognitive performance [46]. There would also appear to be little benefit in extending testing to younger participants since CFQ scores are typically higher, rather than lower, before the age of 60 [9, 12]. However, additional investigation is required because none of these studies determined specific liabilities or controlled for depressive symptomatology. Although the CFQ was not specifically designed to detect cognitive failures in old aged participants an association between CFQ scores and longitudinal cognitive decline has been demonstrated [47]. This may be an especially interesting direction for future research.

A further interesting outcome concerns the relationships among objective cognitive and mnestic functions and CFQ subscales. There were no significant associations (partial correlation coefficients were computed to control variation in depressive symptoms) between any aspect of cognitive failures and general cognitive efficiency (i.e., MMSE score). This is consistent with the definition of the cognitive failures construct i.e., that individuals possess sufficient ability to perform everyday tasks smoothly [2]. In contrast, although digit span was not correlated with total CFQ scores, it was significantly associated with some CFQ factor scores. Specifically, there were significant negative correlations between forward digit span and the CFQ Distractibility and Blunders factors. This may reflect a shared sensitivity to lapses in attention among these measures [34, 48]. Both measures of working memory were also negatively correlated with age.

It must of course be recognized that this study was not without limitations. The CFQ was not specifically developed to assess cognitive failures in older people and requires participants to recall failures over the previous 6 months. It is possible that the impact of ageing might be more evident on different or more restricted sets of CFQ items than those associated with the CFQ factors reported here. Reports of differential changes in cognitive failures [16, 49], including the present study, highlight the need for further research. The suitability of the CFQ for this purpose may be questioned. It is unlikely that participants of any age can recall every minor cognitive failure over the preceding 6 months and this issue may be more pronounced in older individuals [50]. For the present study, the most serious consequence of recall bias would be if it was unequally distributed across the age groups or different social contexts. As a source of measurement error, recall bias may also have hindered detection of meaningful effects. A potential solution to both problems would be to record cognitive failures immediately, or soon after, their occurrence over an appropriate time period, as has recently been reported [51].

To conclude, previous research about cognitive failures in ageing is limited by an inability to determine whether the reported outcomes were due to variation in depressive symptomatology. This potentially serious confound clouds understanding of the true relationship between aging and cognitive failures. In the present study, we found no discernable effect of age on cognitive failures in cognitively healthy elders regardless of variation in depressive symptomatology. In contrast, differences in cognitive failures varied between individuals from distinct social contexts. Specific types of cognitive failure were differentially associated with depressive symptoms and were differentially influenced by social context.

## Supporting information

S1 File General data. (XLS)

S2 File CFQ in detail. (XLS)
